# Upcoming Transformations in Integrated Energy/Chemicals Sectors: Some Challenges and Several Opportunities

**DOI:** 10.1021/acs.jpcc.2c05192

**Published:** 2022-12-16

**Authors:** Alberto Striolo, Shanshan Huang

**Affiliations:** † School of Chemical, Biological and Materials Engineering, University of Oklahoma, Norman, Oklahoma 73019, United States; ‡ Department of Chemical Engineering, University College London, London, U.K. WC1E 7JE; § Applied Sciences, Innovation and Engineering, BP International Ltd., Sunbury-On-Thames, U.K. TW16 7LN

## Abstract

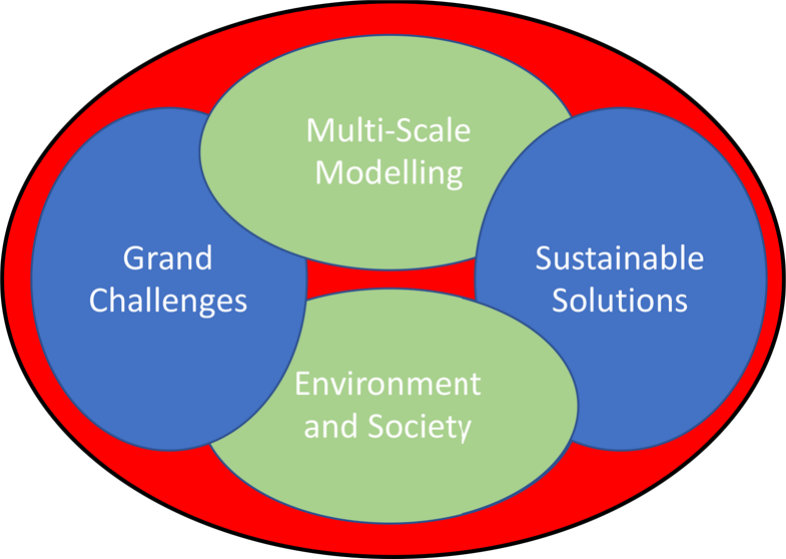

The sociopolitical events over the past few years led to transformative changes in both the energy and chemical sectors. One of the most evident consequences of these events is the significant focus on sustainability. In fact, rather than an engaging discussion within elite social circles, the search for sustainability is now one of the hard requirements investors impose on companies. The concept of sustainability itself has developed since its inception, and now it encompasses environmental as well as socioeconomic aspects. The major players in the energy and chemical sectors seem to embrace these changes and the related challenges; in most cases, tangible ambitious goals have been proposed. For example, bp aims “to become a net zero company by 2050 or sooner, and to help the world get to net zero”. Although tragic events such as the war in Ukraine directly affect global supply chains, leading to some reconsiderations in medium-term industrial and political strategies, trends and public demands seem determined to pursue ambitious sustainable goals, as tangible as the European Union’s “Fit for 55” climate package, approved on May 12, 2022, which effectively bans internal combustion engines for new passenger cars and light commercial vehicles from 2035. These trends will likely lead to profound changes in both the chemical and energy sectors. While some predictions may miss the target, speculating about upcoming challenges and opportunities could help us prepare for the future. This is the purpose of this brief Perspective.

## Introduction

Both the energy and chemical sectors face unprecedented challenges and a very uncertain socioeconomic landscape. The COVID-19 pandemic had a strong effect in lowering, in the short to medium term, the global demand for fuels and some commodities, although selected specialty chemicals were in high demand during the pandemic.

Acute environmental awareness is now reshaping major consumer sectors; for example, the automotive industry is poised to see growth of shared ownership, automation, and electrification. The resultant reduction in demand for gasoline will affect the future of refineries.^1^ In the United Kingdom, sales of new gasoline/diesel cars will be banned by 2030,^2^ and the European Union will follow suit, having recently banned internal combustion engines for new passenger cars from 2035.^3^ Research investments in refineries will continue, at least for some time, with the expected bulk of the investment targeting mostly carbon capture and hydrogen electrolysis until 2050, with perhaps only 5% of the investment on improving plant efficiency.^4^

Although the demand for jet fuels will likely remain high, the reduction in refining capacity will have a domino effect on the availability of raw materials for the petrochemical industry. Compounded by increased consumers’ requests for bioderived materials, these changes will encourage industry to invent alternative processes to satisfy the global demand for specialty chemicals such as surfactants. Perhaps, in the medium term, the end of the availability of cheap hydrocarbon-based raw materials will allow alternative bioderived products, often considered more expensive, to become competitive.^5,6^

Even though widespread adoption might be delayed by the current high inflation rates,^7^ producing specialty chemicals from bioderived sources, combined with the expected energy transition, will contribute to high levels of decarbonization, required to achieve both the 2015 Paris Agreement and the Sustainable Development Goals put forward by the United Nations. Achieving these ambitious goals is critical for the survival of individual companies, if not of entire industrial sectors. It is perhaps telling that the concept of sustainability itself has evolved since its inception, and from a discussion piece in intellectual circles, it has become a yardstick for which boards of directors are expected to measure up to.^8^

It is also telling that the major energy companies recognize and align their strategies with these needs. According to their websites, bp aims “to become a net zero company by 2050 or sooner, and to help the world get to net zero”;^9^ Chevron strives “to protect the environment, empower people, and get results the right way”;^10^ for Saudi Aramco “the circular economy is a pragmatic concept that can provide direction for a sustainable future”;^11^ ExxonMobil is “committed to producing the energy and chemical products” needed by our society while “protecting our people, the environment and the well-being of the communities where [they] operate ”;^12^ Shell aims to “reduce the carbon intensity of the energy products [they] sell by 100% by 2050”;^13^ TotalEnergies has the ambition to “be a world-class player in the energy transition”;^14^ and, for ENI, “sustainability means contributing to a socially just energy transition that guarantees access to energy for everyone, while protecting the environment”.^15^ While these statements respond to environmental regulations,^16^ achieving such goals requires overcoming several multilevel hurdles: for example, when communities display economic optimism toward the fossil fuel industry, they tend to support the status quo;^17^ environmental regulations can yield different outcomes on different sectors;^18−20^ sustainable strategies require stable environmental regulations to be effective;^21^ and developing new bioderived commodities necessitates compromises regarding the use of arable land and other resources.^22^ These examples show that technological innovation is more and more entangled with social perception, economics, and policy making.

**Figure 1:**
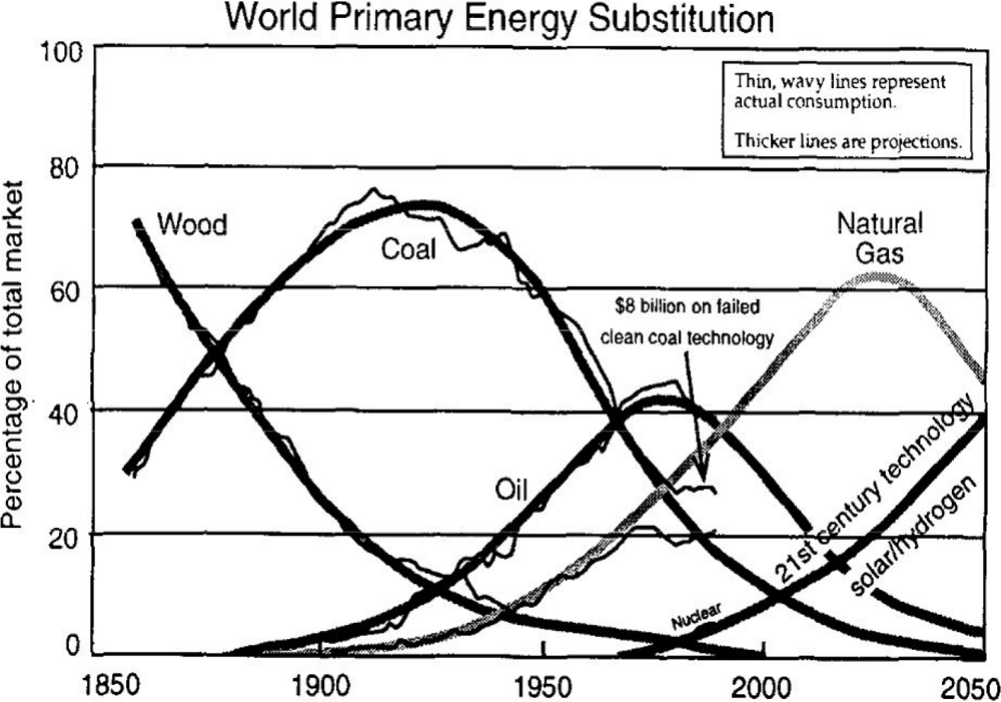
Schematic of past and projected energy source transitions, indicating the trend from solid (wood and coal) to liquid (oil), to gases (methane and now hydrogen). Reproduced with permission from ref (25). Copyright 1995 Elsevier.

While the challenges faced by the energy and materials sectors are not trivial, the chemical engineering profession is known for its ability to rapidly adapt and innovate.^23^ For example, via the entropy generation minimization approach, process engineers achieved savings in CO_2_ emissions in excess of 15%.^24^ It is widely recognized that this profession has enabled several energy transitions in the past, as shown schematically in Figure 1.^25^

However, to continue to succeed, we need to prepare for the imminent challenges.

**Figure 2:**
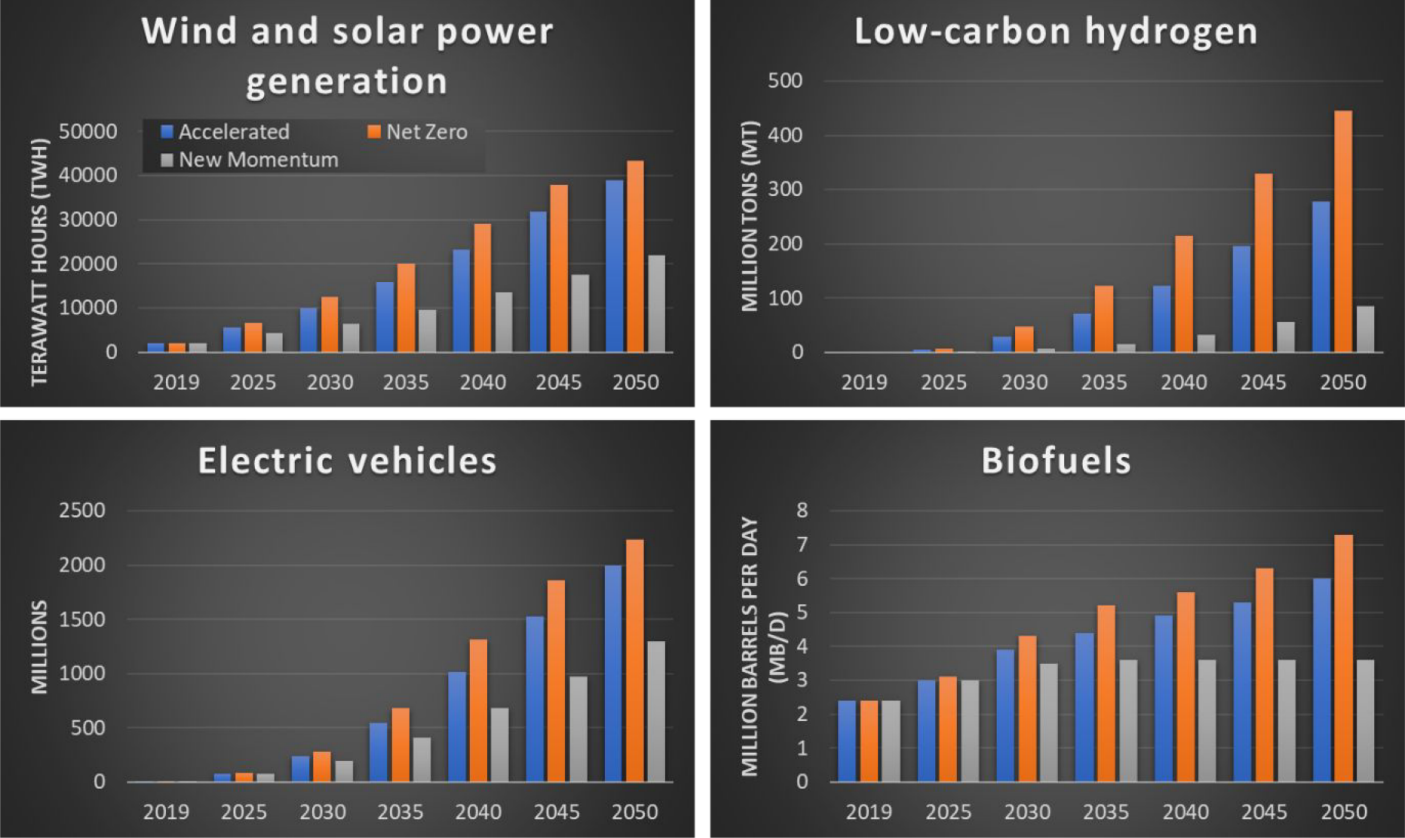
Projected amount of energy generated by solar and wind (top left) and biofuels (bottom right); projected millions of electric vehicles sold (bottom left) and (top right) projected demand for low-carbon hydrogen (blue and green) to 2050 according to three scenarios related to the reduction of CO_2_ emissions: accelerated (blue bars), net zero (orange bars), and new momentum (gray bars). Data extracted from ref (26).

**Figure 3:**
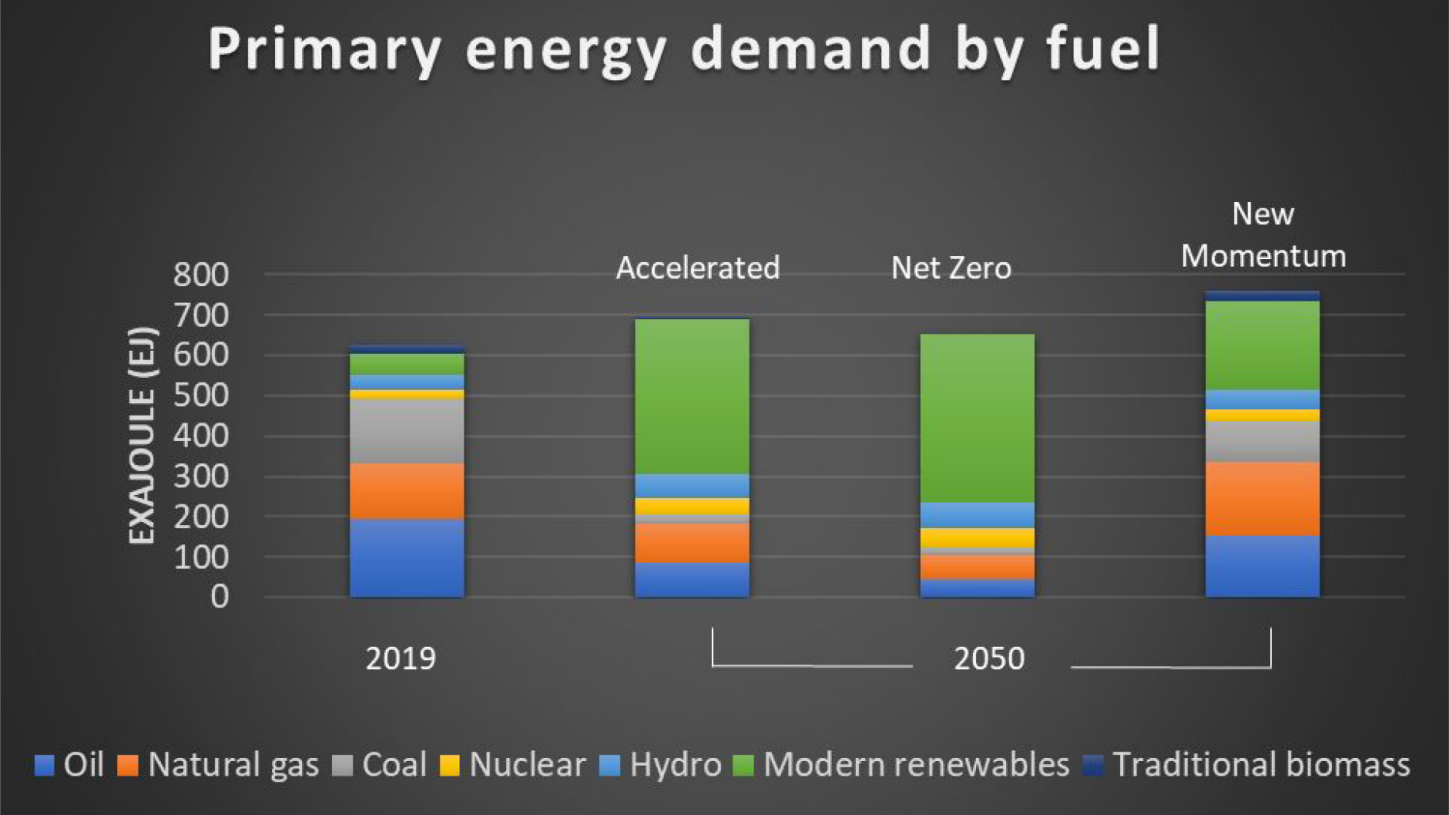
Primary energy demand by fuel: 2019 data compared to 2050 predictions for accelerated, net zero, and new momentum scenarios. Modern renewables include wind, solar, geothermal, biofuels, biomethane, and modern biomass. Data extracted from ref (26).

In order to prepare, the analysis shown in Figure 1 could be contextualized with recent energy outlooks.^26^ For example, to explore possible implications of the energy transition, bp considered three scenarios, identified as “accelerated”, “net zero”, and “new momentum”, toward exploring possible implementations of existing technologies to reduce CO_2_ emissions. Of note, the report was developed before the start of the 2022 war in Ukraine, and it did not contemplate emergent technologies. The implementation of existing low-carbon technologies is expected to enable a decrease in the share of fossil fuels and an increase in renewable energies as primary sources, combined with an increase of up to ∼50% of final energy consumption in the form of electricity by 2050 (see Figure 2). Of note, the accelerated and net zero scenarios expect that ∼90% of the new vehicle sales will be pure battery electric or plug-in hybrids by 2050. In the same time frame, the demand for low-carbon hydrogen is expected to increase to 280–450 mt per annum to satisfy difficult-to-electrify sectors such as iron and steel manufacture. An increase of the share of renewables as primary energy sources for more than 50% is expected by 2050 in the accelerated scenario, which will lead to a diversification of the fuels available in the market. Nevertheless, even though the share of fossil fuels will decrease during the transition, the growth in standards of living, combined with population growth, will lead to an increase in primary energy demand. This is shown in Figure 3, where the portfolio expected by 2050 is compared to that of 2019: the use of coal will likely decrease, but that of natural gas can increase in the same time frame.

To prepare for the upcoming transformations, chemical engineering education will have to rapidly adapt, potentially embracing and leveraging new complementary disciplines. Already in 2007, Prausnitz warned that, even though the task of chemical engineers is to advance knowledge and invent/improve product and processes, in the postmodern world, these tasks cannot be achieved without paying attention to cultural needs, which include sustainability.^27^ Focusing on the energy transition and related challenges, it is important to recognize that environmental implications differ country by country, as local decisions strongly depend on “energy endowments”. Traditionally, for example, the electricity ladder follows a somewhat prescribed path, which starts from fossil fuels and gradually transitions to nuclear and renewables.^28^ To handle geographical and societal differences, we advocate that elements related to social studies, environment, and economics should enrich chemical engineering education.

The remainder of this Perspective summarizes a few possible research and educational activities that could address future challenges and opportunities in the materials and energy sectors. These few examples primarily reflect the research interests of the authors and provide broad perspectives. It is hoped that individual researchers will use these examples as possible inspiration rather than as firm guidelines for future research.

## Some Research Opportunities

**Figure 4:**
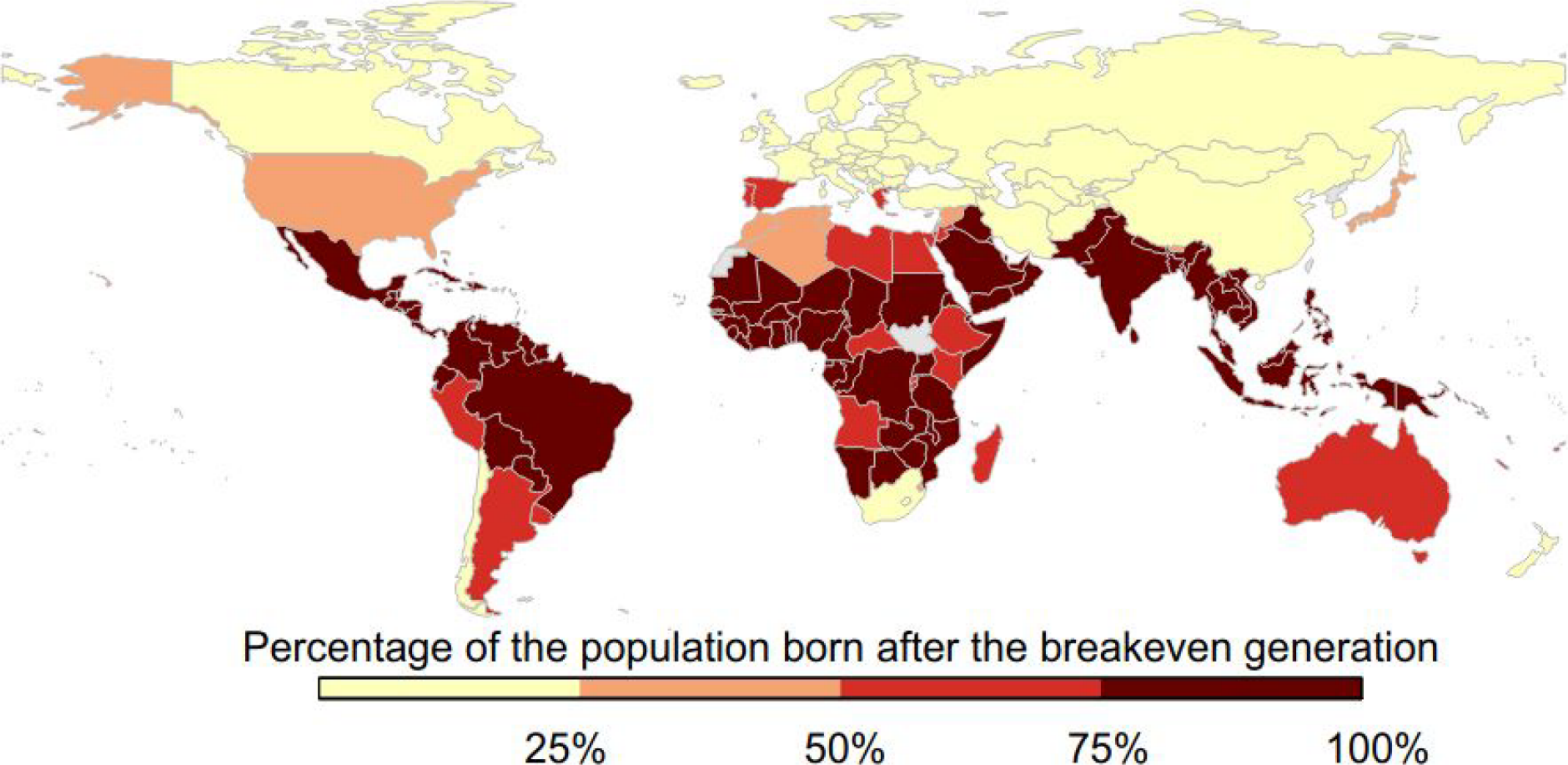
Percentage of the existing population born after the break-even generation according to the “short-lived” net benefits as estimated by Yang and Suh. Generations born before the break-even generation will experience net economic losses due to climate mitigation policies, while generations born after the break-even generations will experience various levels of economic benefits. The results show a strongly heterogeneous distribution across the world. Reprinted with permission from ref (51). Copyright 2021 Springer Nature.

Building upon the tradition of successful impact due to fundamental research (e.g., the internet is the result of U.S. Department of Defense investments in the 1960s), it is argued that decisive research investments will allow the community to achieve the goals of the 2015 Paris Agreement.^29^ Quantitative analysis of various pathways to achieve such goals indicate that it will be essential to decarbonize the production and utilization of energy.^30,31^ While the estimates for the investments needed vary widely,^32,33^ according to Andrijevic et al.,^34^ such investments would amount to just a small fraction of those injected by worldwide governments in response to the COVID-19 pandemic [∼USD 12 trillion in October 2020]. To put these sums in perspective, as of March 2022, estimates of the cost of U.K. Government measures in response to the COVID-19 pandemic range from £310 to £410 billion,^35^ while the U.S. Inflation Reduction Act of 2022^36^ has earmarked $369 billion for investments in “energy security and climate change”. It also helps to remember that several perspectives,^37^ including national policy documents from the United States,^38^ the United Kingdom,^39^ and the European Union,^40^ manifest concerns regarding costs related to climate policy. These concerns should however be considered in a wide context. Koberle et al.,^41^ for example, reviewing the relevant literature,^42−44^ concluded that cost estimates related to climate mitigation do not include the economic benefits of avoided impacts. In general, the estimated costs due to climate change mitigation are based on the gross domestic product (GDP) loss compared to no mitigation, which has been estimated as 2–6% of global GDP by 2100.^45^ On the other end, the avoided economic losses achieved by stabilizing the global temperature at 2010 levels^46^ have been estimated in 23% of the global GDP by 2100.^47^ A consistent framework for comparing costs and benefits could provide benchmarks for reframing climate policies, for example, using “carbon dividend” instead of “carbon tax” terminology,^48,49^ thereby achieving effective public communication.^50^ In this landscape, Yang and Suh conducted an interesting analysis regarding the lifetime costs and benefits of age cohorts across countries.^51^ The results show that, in general, generations born after 1960 will gain benefits from climate change mitigation in lower income countries, while in many high income countries the percent of the existing population expected to enjoy net economic gains from climate mitigation policies is low (see Figure 4). These results are important for appreciating the challenges in building consensus for climate policies, which we believe is essential to achieve ambitious common goals.

Innovation occurs continually across the energy and materials industries, and it ranges from incremental improvements to disruptive discoveries. In recent years, strong emphasis has been placed on data-driven innovation, building on the enthusiasms gathered by initiatives such as “Industry 4.0”, as well as by success stories related to artificial intelligence (AI),^52,53^ often via its machine learning (ML) offspring. This is evident from press releases. Chevron, e.g., claims to use “AI technology and data analytics to drive logistics, increase efficiencies and lower costs”.^10^ Ahmad A. Al-Saadi, Senior Vice President of Saudi Aramco, stated that, “Nations who understand the power of transforming data into useful knowledge will enjoy a strong and prosperous future.”^11^ But how will the impressive developments in AI allow the energy and chemical sectors to address the technical challenges posed by their efforts to enable more sustainable operations? Some technical challenges (e.g., asphaltene precipitation and hydrate formation) have affected industry for decades; while ML predictions could help mitigate some of the risks associated with these phenomena, an exquisite fundamental understanding, at the molecular level, of the chemical mechanisms at play remains critical to preventing the related environmental and safety risks. Nevertheless, in the near future it will be possible to harness ML, integrate it with a variety of cutting-edge multiscale computational approaches (some of which are already being implemented), and innovate various aspects and processes relevant for both the energy and chemical sectors.^54^ Of particular importance is the development of deep learning techniques for preventing and mitigating cyber attacks.^55^

**Figure 5:**
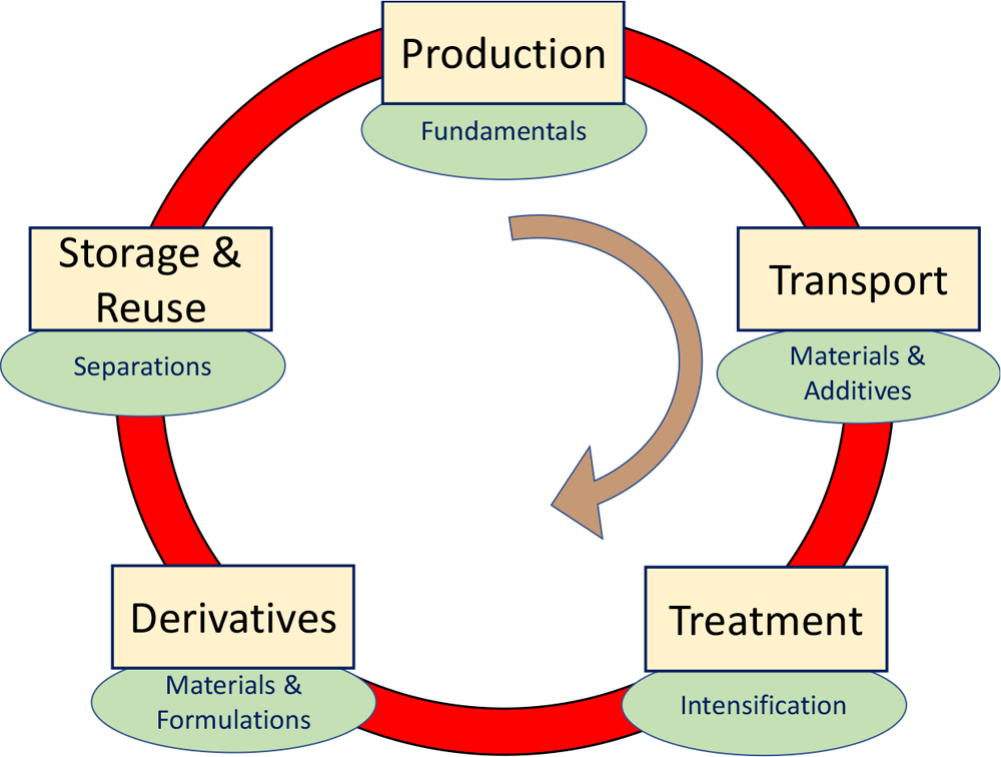
Schematic representing a drastically simplified view of the integrated energy and materials industries, in which the concept of circular economy is borrowed and integrated within the carbon cycle, from hydrocarbon production, to storage, and reuse of the final products, including CO_2_ geological sequestration. The ovals identify possible fundamental research topics for each stage.

To guide the discussion beyond AI, Figure 5 provides a drastically simplified schematic for the integrated energy and materials industries. This simplified view embeds the financial concept of circular economy,^56^ as exemplified by the carbon cycle. In the traditional implementation, the journey begins with the production of hydrocarbons. Once extracted, the hydrocarbons are transported to where they are used (e.g., energy production) or where they are transformed into useful chemicals. The next step is the refinement, which includes processing facilities such as natural gas processing plants. This stage is essential for transforming hydrocarbons into derivatives, specialty chemicals, plastics, and commodity products used worldwide. One such product is isopropyl alcohol, used to prevent the spread of viruses such as COVID-19; another is poly(ethylene), which accounts for over 30% of the plastics used worldwide in bags, bottles, hip replacements, etc.^57^ To reduce the environmental impact, these products should be reused, recycled, and, only when no other use is possible, disposed of appropriately. This is not easy. Already in 2009, Hopewell et al.^58^ discussed some of the challenges faced by those who seek to recycle plastics. The authors noted that ∼4% of world oil and gas production is used as feedstock for plastics, and that plastics are the raw materials for many disposable objects we, as a society, enjoy and discard within a year since they have been produced. To recycle these materials, one needs to recognize that different materials have different properties: thermoplastics (e.g., PET, PE, and PP) can be mechanically recycled, while thermosetting polymers (e.g., epoxy resins) cannot be recycled so easily. Some plastics might contain catalysts or dyes, which would make it difficult for such materials to be reused. Hence, while much current research efforts focus on the “upcycling” of polymers,^59^ polymeric materials frequently undergo secondary recycling, according to which they are used in products that do not require high-quality materials, or quaternary recycling, according to which they are used for energy recovery. One alternative is ternary recovery, in which the plastics are depolymerized to their initial constituents.^60^ To identify the optimal solutions, it would be effective to implement the cradle-to-cradle principle in designing plastic materials^61^ and to consider, already at the design stage, the processes by which polymers can be collected, separated, treated, and reused after their primary use.

At the end of the cycle in Figure 5, after products are used, and potentially reused multiple times, different fluids and gases are generated, one of which is CO_2_. These byproducts need to be handled consciously to achieve the goals of the 2015 Paris Agreement, and several strategies have been mapped accordingly.^62^ These strategies require various combinations of low-carbon-energy supplies, reduced energy use, efficient operations, and CO_2_ removal. Although variations exist, all approaches identified to achieve the goals of the Paris Agreement require long-term sequestration of CO_2_ via geological repositories.^63^ Although this final stage closes the carbon cycle initiated with hydrocarbon extraction, it should be noted that, in the realization shown in Figure 5, the cycle would only close after millions of years, when and if the sequestered CO_2_ is reduced to hydrocarbons.^64^

In each of the stages broadly identified in Figure 5, aggressive research and development programs have contributed to the long-term success of both the energy and chemicals industries for many generations. Prior innovations have led to, e.g., identifying previously unknown hydrocarbon reservoirs,^65^ producing unconventional hydrocarbons such as shale gas,^66^ reducing the risks associated with production^67^ and transport,^68^ new energy-efficient separation processes (e.g., membrane separations),^69,70^ new catalytic processes,^71^ and process intensification.^72^ Despite frequent changes in priorities due to the fast-changing socioeconomic landscape, as demonstrated by the high level of attention to hydrogen production and utilization that has affected the energy industry over the past 12–24 months,^73^ research in the various stages summarized in Figure 5 remains critical. Some possible examples, which reflect the authors’ current personal interests, are listed in Table 1. These examples focus on computational approaches, although there is no doubt that technological progress can only be achieved when experiment, theory, and computation are joined synergistically.

**Table 1:** Examples of Research Projects Which Could Benefit Individual Stages of the Simplified Integrated Energy and Chemical Sectors Shown in Figure 5

CO_2_-Based Enhanced Recovery for Unconventional Reservoirs. Relevant Stage in Figure 5: Production
**Problem:** Hydrocarbons could be trapped within porous matrixes with multiple porosities. Because viscous hydrocarbons can be difficult to produce from formations characterized by narrow pores, they could remain in place within nearly depleted reservoirs. Further, existing hydrocarbon deposits could contain large amounts of CO_2_ or other gases. It is desired to produce these hydrocarbons, to both enhance production and reduce the likelihood of possible future leaks, should the formations be used for other purposes, for example geological storage of greenhouse gases.
**Potential solution:** Among various enhanced recovery approaches, it would be desirable to produce the trapped hydrocarbons while sequestering more CO_2_. In fact, CO_2_ has been used for enhanced oil recovery,^74^ and pilot operations show that this process offers an attractive solution for sequestering CO_2_ captured, for example, from power plants.^75^ CO_2_, via dissolving within the hydrocarbons, could also reduce their viscosities. It is also possible to investigate how other gases, such as low molecular weight hydrocarbons, could be used for enhancing recovery of the viscous high molecular weight compounds.^76^
**State of the art and fundamental challenges:** This approach is currently applied in several field sites, for example related to geological storage of CO_2_.^77^ Nevertheless, to increase efficiency, it is required to quantify phenomena that occur within porous systems and change fluid properties significantly compared to bulk counterparts. Once quantified, these properties could be included in reservoir simulators to predict and derisk the future development of a petroleum reservoir. Some of the key physical phenomena that need to be quantified include, but are not limited to, the following: (1) As the brine composition changes upon dissolution of both CO_2_ and hydrocarbons, density, viscosity, and other physical properties also change. (2) As the CO_2_ sweeps the porous formation, hydrocarbons could remain trapped in some of the pores. (3) Confinement in tight pores could affect thermodynamic and transport properties of the individual components within the complex multicomponent mixtures. (4) Wetting of the minerals depends on system composition, surface roughness, and heterogeneity. (5) Sequestered CO_2_ could migrate and eventually be released.
Fines Migration in Geological Formations. Relevant Stages in Figure 5: Production and Storage
**Problem:** As fluids transport through porous matrixes, particulates (fines) break free from the rocks, become mobile, and might aggregate, potentially blocking pores, changing the preferential transport pathways, and reducing the permeability of a formation. This could reduce the productivity of conventional and unconventional hydrocarbon formations. The problem is likely to occur also for geological CO_2_ and H_2_ storage processes, where it could compromise the storage capacity and impair injectivity, especially for liquid fluids.
**Potential solution:** To solve this multiscale problem, it will be necessary to quantify the effective particle–particle interactions as a function of system conditions,^78,79^ the mechanical properties of the particles themselves, including elastic deformation, the effect of fluid flow, the transport of the particles through the matrix,^80^ their agglomeration into aggregates,^81^ their deposition,^82^ and eventually the effect of particle agglomeration on the permeability of the host materials.
**State of the art and fundamental challenges:** Current approaches rely on periodic treatments of the geological formations, which in oil fields cause delays in oil production. The technical challenge in developing predictive capabilities, which would be helpful, for example, for better planning treatment processes of the formations, consists in taking into consideration phenomena that occur over different time scales. For example, molecular simulations at the atomistic resolution are appropriate for discovering subtle features related to particle–particle interactions but cannot describe the length and time scales involved in particle migration through a pore, for which Lagrangian approaches are better suited. Taking into consideration this technical hurdle, several fundamental phenomena are relevant, including the following: (1) Understand how fines form, depending on fluid transport rates and fluid composition. (2) Quantify the effect of heterogeneity on particle transport through pore matrixes. (3) Predict particle aggregation when the effective interactions are highly anisotropic. (4) Quantify particle deposition, aggregation, desorption, disaggregation, and the resultant effects on permeability. (5) Properly account for the elastic deformation of the particles. Modeling efforts,^82,83^ at the appropriate length scales and validated by cutting-edge experiments, can address each of these phenomena. Stochastic kinetic Monte Carlo approaches^84,85^ may provide the tool to simultaneously take into consideration phenomena that occur at different times.
Refining On Demand. Relevant Stages in Figure 5: Treatment and Derivatives
**Problem:** As the demand for gasoline is expected to decrease in the near future, the demand for jet fuel and other specialty chemicals could remain substantial and perhaps increase. The design of existing refineries will have to change to accommodate for lower volumes and perhaps different feedstock (e.g., biofuels). Because the jet fuel and specialty chemicals demand will vary geographically, e.g., via proximity to airports, the supply chain will have to adapt to provide just-in-time resources where needed.
**Possible solution:** Process design will have to consider different scenarios, to accommodate for various feedstocks and product lines, taking into consideration both capital and operating costs, as well as environmental impact and societal preferences. The most attractive solution should be selected based on a compromise quantified by life cycle analysis. It is likely that process intensification will offer several advantages.^86^ In response to local demands, new processes will be needed to accommodate location-specific raw materials; e.g., biomass pyrolysis has led to fungible biofuels.^87^
**State of the art and fundamental challenges:** Commercial opportunities are arising for producing jet fuel using sources alternative to fossil fuels,^88,89^ and airlines are eager to lower the environmental footprint of the sector.^90^ As the size of the chemical plants reduces, new technologies will be required to reduce emissions and energy consumption. Because the raw materials change, thermodynamic properties and data will be needed to optimize processes via process design software. Perhaps, ML will have the opportunity to estimate the thermophysical properties needed for some of the compounds.^91^ Also, given that the size of the plants will be smaller than those in current use, it might be both a challenge and an opportunity to test relatively new technologies such as microchemical processing systems, including distillation columns on a chip,^92^ providing the volumes of jet fuels and specialty chemicals required in a given location, while maintaining costs within acceptable limits. Fundamental challenges that will need to be addressed include, but are not limited to, identifying appropriate scale-up approaches, and better understanding of the separation processes, as the reduced sizes of the equipment could enhance the importance of interfacial effects such as capillary forces, generally ignored in the design of large-scale processes such as distillations. New separation technologies, for example, membranes,^68^ could find application, which will reduce the energy footprint.^93^
CO_2_ Transport via Hydrates. Relevant Stages in Figure 5: Transport and Storage
**Problem:** Carbon capture and sequestration projects tend to focus on large single-point CO_2_ emitters, where carbon capture is technically viable. However, many midsize emitters exist. How can we make it viable to capture CO_2_ from these disperse sources and transport it to geological repositories in a way that is economical and environmentally sound?
**Possible solution:** Clathrate hydrates could be used to capture CO_2_ and transport it. The process does not require high pressures or very low temperatures, and hydrates could be stable at near-ambient condition during transport. Transporting hydrates implies transporting large amounts of water as well, but the hydrates themselves could be used to achieve long-term CO_2_ sequestration.^94^ Because hydrate-based technologies have also been proposed for water purification applications,^95^ there is an opportunity to capture CO_2_ using hydrates, transport the hydrates to locations where CO_2_ is to be sequestered, and recover purified water when the hydrates are dissociated.
**State of the art and fundamental challenges:** The stability of CO_2_ hydrates at conditions mimicking deep oceanic sediments has been recently demonstrated in the laboratory,^96^ and progress has been made in the quantification of the fundamental properties of CO_2_ hydrate formation and morphology.^97^ Although these studies, and upcoming field tests, are related to sequestration, a recent techno-economic analysis suggests that using hydrates for CO_2_ capture could be attractive commercially.^98^ For clathrate hydrates to be technologically viable in the transportation of gases, it is necessary to increase formation and decomposition kinetics, which are slow because of the self-preservation effect.^99^ It is also necessary to enhance the stability of the hydrates at near-ambient conditions during transportation to reduce the transportation costs.^100,101^ To overcome these technical challenges, it is necessary to develop a deep understanding regarding the molecular mechanisms responsible for the nucleation and growth of clathrate hydrates, while taking into consideration transport mechanisms, as well as a number of features of clathrate hydrate particles, including, but not limited to, their wetting properties.^102,103^ Both thermodynamic^67^ and kinetic promoters would be needed for such purposes. If one were to pursue the possibility of combining CO_2_ capture and transport with water purification, such promoters would need to be environmentally benign.
H_2_ Transport. Relevant Stage in Figure 5: Transport
**Problem:** Hydrogen promises to decarbonize many sectors, including hauling and energy, and it also promises to connect different parts of the energy sector.^26^ The >100 million metric tons of H_2_ used each year are used mostly for oil refining and ammonia manufacture, and it is not transported for long distances. To achieve its full potential, H_2_ will need to be mobile. However, several problems need to be overcome, ranging from metal embrittlement^104^ to environmental leaks.^105^ In fact, the environmental footprint of blue hydrogen strongly depends on the leaks during production and transport.^106^
**Possible solution:** Polymeric pipelines could be used to transport some of the H_2_ across medium distances. It is expected that H_2_ will not be very soluble in the polymers, but because it is a small molecule, its diffusion through the polymers could be fast, leading to unacceptable leaks. It might be possible to use copolymers and/or polymeric blends to reduce the free volume available for H_2_ molecules to adsorb within the polymer, thus reducing the amount adsorbed, while also making it more difficult for the H_2_ molecules to diffuse. It might also be possible to design coatings for the pipelines to introduce an additional barrier to H_2_ transport. The latter might also be applicable to metallic infrastructure and perhaps reduce the changes of embrittlement. Other solutions include the use of chemical carriers, e.g., ammonia.^107^ This possibility is discussed in Table 2.
**State of the art and fundamental challenges:** Tests have been conducted for H_2_ transport in pipelines originally installed to transport natural gas,^108^ and best practice recommendations have been provided based on the results. Going forward, the mechanisms of interactions and transport for H_2_ through polymeric materials need to be quantified, as prior work focused on heavier molecules.^109^ It is known that interactions involving H_2_ strongly depend on system conditions, with quantum effects becoming important and sometimes dominant at low temperatures.^110^ Thus, from the modeling point of view, it will be necessary to maintain adaptability for any prediction to the practical conditions of pipeline use. It will be challenging to test whether the models developed for widely used polymers, such as poly(ethylene), as well as composite materials,^111^ will be reliable for predicting their performances for H_2_ containment. It is possible that, because of its small molecular size, H_2_ transports preferentially along crystal–amorphous polymer interfaces, which could be used to control H_2_ leaks when multiscale models can predict the structure of multicomponent systems containing coatings and fillers, as well as different polymer phases. Because the H_2_ molecule is very small, one of the main challenges is to achieve atomic-size resolution in the predictions while maintaining a reliable description of the macroscopic properties of a polymeric material.

However, would these rather conventional lines of research be sufficient to enable the successful transition of these sectors to a more sustainable future?

It is argued here that responding to the societal quests for sustainable development implies major shifts in the structure of both the energy and materials industries. This is perhaps more strongly evident in two examples: (1) the sensational push for enabling the hydrogen economy and (2) the implementation of circular cradle-to-cradle concepts within the manufacture of new materials.^112−114^ Both examples could be seen as attempts to shorten the realization of the circularity in the carbon cycle. Perhaps, then, one could extrapolate and suggest that the transformation of the energy and materials industries we are witnessing could substitute the giant cycle summarized in Figure 5 with several smaller cycles, adapted to local realities. Such local solutions would differ depending on the availability of resources and the local demands; as these local solutions would vary with time, a truly agile industry will be necessary. Perhaps tools such as those developed for the optimization and scheduling of industrial processes^115−117^ could be helpful in guiding the implementation of this new realization of global industries. With this in mind, in Table 2 some research opportunities which are more local compared to those discussed in Table 1 are presented. The circular element is discussed explicitly in Table 2, to highlight how fundamental research could allow the community to achieve sustainable development.

**Table 2:** Example of Research Projects That Could Enable Us to Achieve Sustainability via the Implementation of the Circular Concept within the Integrated Energy and Chemical Sector

Optimization of Geothermal Energy Production
**Problem:** Geothermal energy promises to provide support as baseline energy; however, only a few commercial projects exist worldwide. The initial high capital investment required for drilling the wells could be compromised when the permeability of the subsurface is not maintained and when naturally occurring radioactive materials are present at too high concentrations. These uncertainties make new projects very risky.
**Possible solution:** Fundamental studies on outcrop rock samples could help identify fluid compositions that are not conducive to loss of productivity during production.^118^ The aqueous composition should be quantified, as it could offer the opportunity of recovering rare earth minerals or other components such as lithium, which are currently in high demand to achieve sustainable development. Several direct lithium extraction technologies have been proposed,^119^ e.g., solvent extraction, adsorption, ion exchange, and membranes. Some of these technologies are being tested in the field.^120,121^ An alternative approach for geothermal energy production is represented by the closed-loop system technology;^122^ to reduce the initial capital investment as well as uncertainties concerning formation permeability, it has been proposed to repurpose retired oil wells.^123^ In the latter case, the thermal gradient might not be very large, yet it might be sufficient to provide base-load energy. These alternative approaches are being tested in the field.
**Fundamental challenges:** Producing single components of the purity and in the amount sufficient to generate commercial interest will require advanced processes and materials for the selective extraction of the desired components. For example, lithium is present in geothermal brines together with Na^+^ and Mg^2+^, which are typically in much higher concentrations.^118^ The new processes need to be effective in the time scale consistent with geothermal energy production (i.e., solar evaporation is likely not feasible), and therefore there is a need for new materials, e.g., Chevrel phase materials.^124,125^
**Circular aspect:** The Li^+^ ions produced from geothermal brines could be used for the fabrication of batteries used to store part of the geothermal energy produced. The circularity is also embedded in the concept that a potential waste (the impurities present in the brines) is turned into profit and used for advanced applications, thus extending and derisking the capital investment made in the geothermal operation. As it has been shown that the length of the geothermal operation is one of the most important parameters that dictate the environmental impact of the technology,^126^ the research suggested here could help reduce the environmental footprint of the operation. In the future, batteries based on other ions (e.g., Zn) could also become attractive,^127^ and the circular approach just mentioned would still be applicable.
Geological H_2_ Storage
**Problem:** Should large amounts of green hydrogen be produced using intermittent energy sources such as solar, temporarily storing the produced H_2_ before it can be used in a variety of technical applications might be required. Because the H_2_ molecule is extremely small, in the gas phase at ambient conditions, and, depending on conditions, reactive, storing large amounts without dispersing it in the environment is technically challenging.
**Possible solution:** Depending on the amount of H_2_ that is required to store, several technologies have been explored, from sorption in porous materials^128^ to the formation of metal hydrides.^129^ Large amounts of H_2_ could be stored in geological repositories.^130^ Muhammed et al. recently reviewed salt caverns and other types of formations in which H_2_ can be stored,^131^ and Williams et al.^132^ estimated the U.K. salt cavern storage potential.
**Fundamental challenges:** While H_2_ storage in salt caverns seems to be largely established,^133^ these formations are not widely available. Hence, fundamental research is required to determine the viability, safety, and reliability of geological storage in alternative formations.^134^ The challenges that need to be overcome are due to the small size and the possible reactivity of the H_2_ molecule, which need to be taken into consideration when taking advantage of the significant expertise developed by the petroleum engineering community via projects for storing gases such as CO_2_ and He. Modeling can be helpful in quantifying the transport mechanisms for H_2_ through various minerals, with the important goal of identifying suitable caprocks to prevent H_2_ dispersion in the environment. Coupled with *ab initio* calculations, reactive force fields^135,136^ could help in understanding the reactivity of H_2_ in various environments, with the goal of identifying minerals that are likely to maintain their barrier properties once exposed to H_2_. It is possible that H_2_ changes rock wettability,^137^ an important parameter in estimating sealing and storage capacities of different formations, and H_2_ can show geochemical reactivity.^138^
**Circular aspect:** The ability of intermittently storing green H_2_ would enable the capture of renewable energy such as solar in the form of chemical energy and use the latter when required. Hence, this circular project could enable the indirect transformation of solar energy to the various forms of energy required by our society. Pilot projects have been attempted,^139^ and they are critical for identifying hurdles that need to be overcome, in addition to the expected fundamental challenges mentioned above. These include, for example, the impact of microorganisms on the feasibility of the project.
CO_2_ Capture and Mineralization
**Problem:** Much focus is currently on containing the CO_2_ concentration in the atmosphere. Carbon capture from concentrated sources is preferred, but research is also considering direct capture from ambient air.^140^ The challenge of the latter is due to the intrinsic low concentration in air, which reduces the driving force for adsorption. New technologies will require processing large volumes of air, and to be effective they will require reducing the energy required for the reactivation of the capturing materials.
**Possible solution:** New advanced materials and processes are needed. Recent emphasis is on metal–organic frameworks,^141^ with interesting twists on the mechanisms used for promoting adsorption,^142^ liquid-infused surfaces to increase the surface area across which gases exchange occurs,^143^ and intelligent process design to speed up carbon capture.^144^ These technologies will need to be scaled up to industrial scale. From the implementation front, it is possible to install direct carbon capture processes in a location where heat is available for the regeneration of the materials, the air concentration is somewhat higher than average, and strategies are available for long-term sequestration.^145^ One example is provided in field installations in Iceland,^146,147^ where direct CO_2_ capture is coupled with carbon mineralization in proximity to geothermal energy production plants. In these processes, captured CO_2_ is mineralized in basaltic formations.^148,149^ To expand the applicability of carbon mineralization processes, it is necessary to identify conditions at which mineralization occurs in the presence of salt water, as opposed to fresh water, which would be a step necessary to deploy the technology in basaltic formations.^150,151^
**Fundamental challenges:** Advancement in the mineralization technology strongly depends on a detailed understanding of reaction mechanisms concerning CO_2_ transformations. Recent advancements in electronic structure calculations as well as machine learning have been significant, and they could contribute to furthering this field with the quantification of environmental effects on reaction mechanisms, identification of suitable catalysts, and determination of the reaction pathways. Because the reactions of interest occur in the environment, at conditions less amenable to be controlled than, for example, within chemical reactors, it will be challenging to predict and control the outcomes of these reactions, but overcoming this challenge has significant benefits.
**Circular aspect:** Being able to permanently sequester CO_2_ would close the carbon cycle and could lead to controlling the properties of carbonated minerals. This could yield new materials, thus enabling advanced applications.
New Transportation Fuels
**Problem:** Toward identifying practical solutions for reducing CO_2_ emissions, much attention is currently focused on H_2_ production.^152^ To reap the expected environmental benefits, it will be necessary to transport the H_2_ to the final users and to use it for either energy generation or chemicals production. However, H_2_ transportation is difficult to accomplish because of the small size of the molecule. In fact, it has been estimated that relatively small H_2_ leaks can negate the benefits expected from “blue” versus “gray” H_2_.^105^
**Possible solution:** One possibility is to convert the produced H_2_ into a chemical carrier. One such candidate is ammonia, NH_3_, which is easier to transport compared to H_2_, although it is toxic and water soluble. Ammonia is currently produced in large quantities via the Haber–Bosch process. Widely used to produce fertilizers, NH_3_ was used already during World War II to power buses in Belgium.^153^ Therefore, one could produce green H_2_ and then use it to produce NH_3_, which could be transported and used in different locations for a variety of applications to achieve economies of scale.^154^
**Fundamental challenges:** Because it is likely that NH_3_ production from green and blue H_2_ will be at a smaller scale than in the current Haber–Bosch processes, it is desirable to identify catalysts that enable the transformation of H_2_ and N_2_ to NH_3_ at moderate pressures and temperatures.^155^ Technological advancements would also be needed to transport NH_3_. Should NH_3_ be used in internal combustion engines, e.g., in maritime transportation,^153^ it will be necessary to improve its ignition features, to optimize engines, as burning of ammonia is slower than that of hydrocarbons, and also to optimize the catalytic converters used to oxidize unburned NH_3_ and reduce NO_*x*_.^156^ Risk assessments are also needed for various final applications involving NH_3_.
**Circular aspect:** Using solar energy to produce the so-called “green” H_2_ via water splitting, then using such green H_2_ for producing NH_3_, and finally using NH_3_ for powering ships with no emissions other than water and nitrogen would provide a complete cycle powered by solar energy, in which the water and nitrogen molecules split at the beginning of the cycle are returned to the environment with no additional emissions.
Bioderived Specialty Chemicals
**Problem:** Both societal demands and environmental regulations are restricting the use of specialty chemical products, e.g., surfactants. New compounds are required that meet or exceed existing performances, while offering a more benign environmental footprint. The problem becomes more challenging when one considers that it is likely that new products will have to be cost competitive with existing ones to be widely adopted.
**Possible solution:** Much attention, as well as significant commercial interest,^6^ is currently focused on sustainable surfactants, which could be biosurfactants such as rhamnolipids, inherently biobased surfactants such as alkyl polyglucosides, or semisynthetic surfactants such as sodium lauryl ethers. Depending on the source, these compounds are produced from fermentation, chemical modification of molecules extracted from plants, and use of biobased chemicals, instead of fossil fuel based raw materials, to produce the specialty chemicals via conventional routes, respectively.
**Fundamental challenges:** The most obvious fundamental challenge is to formulate the new compounds to yield products with high performances. Most specialty chemical products are mixtures that include surfactants and active ingredients. The said mixtures have been optimized, frequently via trial-and-error procedures. Sometimes, even small changes, e.g., a few carbon atoms in the surfactant tail, can compromise the performance of a product.^157^ Therefore, using new classes of surfactants will require developing reliable structure–function relations^158^ to speed up the formulation of the final products. It has been recently pointed out that the properties of biosurfactants can also depend on a variety of factors, including the type and quantity of raw materials available during their production.^159^ It will also be necessary to understand, predict, and ultimately control the interfacial rheology of new products that contain bioderived components,^160^ as this could strongly affect the consumers’ experience and therefore the success of new products introduced in the market. When bioderived products are used as raw materials, it will be important to quantify the environmental footprint compared to that of conventional materials.^161^
**Circular aspect:** This approach offers the opportunity of applying the cradle-to-cradle approach to designing future formulations.^162^ Provided that the entire formulation of the new products enables biodegradation, it might be possible to use chemicals from fermentation production and return them to the environment at the end of their use.
Availability of Critical Mineral Elements for Green Energy Applications
**Problem:** Platinum group elements, Li, and several rare earth elements are essential for solar and wind energy generation, as well as for batteries, which are needed for transitioning to renewable energy.^163^ Out of the many such elements identified by the U.S. Geological Survey,^164^ e.g., Ce, Co, Li, Mn, Pt, Zn, and others, the United States lacks domestic production of 14 and is more than 50% import reliant for many others. It is worth noting that many of these essential elements can be toxic when released in the environment.
**Possible solution:** Many critical elements are present as trace components in a variety of environments, ranging from geothermal brines to produced waters, and from saline lakes such as the Salton Sea, to contaminated bodies of water such as legacy waters and tailings in mining districts.^165^ In locations where passive treatment systems have been installed to achieve environmental remediation, relatively high concentrations of some elements are now available within the residual solids.^166^ Could these systems, which can in some cases be environmental liabilities, be transformed into resources for critical minerals? Further, once batteries and other materials used in renewable energy applications are spent, it would be desirable to extract the critical elements from the spent materials.^167^
**Fundamental challenges:** Engineering solutions are required to be effective in the field, where multicomponent systems are present, where fouling is highly possible, and where conditions can be highly corrosive. The systems themselves can undergo oxidation when exposed to air, and as the composition changes, ionic compounds will show different tendencies to precipitate and/or react. Research is required in materials science,^69^ in process optimization, and in the fundamental understanding of thermodynamic properties of multicomponent aqueous ionic systems. It is likely that the high costs of the needed technologies will affect their field implementation: new economic models are required to quantify environmental and societal aspects in cost–benefit analysis.
**Circular aspect:** The key circularity aspect is represented by the critical elements, which currently represent environmental liabilities in systems such as abandoned mining districts: extracting them could reduce the environmental toxicity of those sites while enabling a transition to the green economy. To ensure that the process is circular, no new environmental harm must be accepted, and new energy storage devices must be designed following the cradle-to-cradle principle,^161^ e.g., in a way in which the device itself enables recovery of the critical elements at the end of its useful lifetime.

To confirm that innovative processes indeed reduce the environmental impact compared to existing processes, it is important to conduct assessments based on the life cycle of the product, service, and/or process, as appropriate. The life cycle assessment (LCA),^168^ which is becoming the prevailing tool for this quantification, considers products from cradle to grave, estimates the environmental impact over a variety of categories, including but not limited to CO_2_-equivalent emissions, and identifies the “hot spots”, which contribute in large part to the environmental impact of a given technology.^125^ Because of these attractive features, LCA facilitates decision-making, although LCA results can be very variable. For instance, the carbon footprint of electricity generated from geothermal energy has been estimated to span from ∼5 to ∼800 g of CO_2_ equiv/kWh.^169^ This variability is due in part due to LCA methodological choices like the definition of the system boundary but also to differing site-specific conditions such as, in the case of geothermal energy production, the composition of the geothermal fluid or the depth of the geothermal reservoir.^170^ To overcome this variability, and to support local decision-making, accurate location-specific data are required. Current research in the field aims at developing simplified models^171^ and in conducting global sensitivity analysis of entire LCA models and background inventories,^172^ which allow for the identification of the most influential parameters in estimating the environmental impact of a technology. Future developments should focus on developing LCA models that account for environmental, economic, and societal impacts of products, processes, and services. It is argued that the life cycle sustainability assessment lies at the intersection among the three aspects.^173^

This need for reconciling aspects related to economics, the environment, and society is strongly reminiscent of Prausnitz’ recommendations from ∼15 years ago,^27^ according to which the chemical engineers of the 21st century should follow not only Athena (representing fundamental science) and Hercules (technological innovations), but also Nausica (societal needs). To achieve ambitious sustainable goals, training and education should also evolve. For example, the energy mix implemented by each country depends on the level of development and, perhaps to a minor extent, on the resource endowments.^174,175^ Jianchao et al.^176^ reviewed policies in place in the G7 countries as well as in China toward promoting the energy transition and found that the approaches vary among these countries, as they depend on the country-specific system, economy, technology, and behavior. New approaches are required to enable a transition that limits global warming, secures socioeconomic development, and promotes social inclusion. Recognizing that these goals need to be harmonized, Vanegas Cantanero,^177^ for example, proposes to adopt existing technologies that improve the efficiency, affordability, and reliability of energy systems (areas where engineers can certainly lead the efforts) while also promoting citizens’ participation in policy making, boosting transparency, accountability, and trust. Clearly, merging elements of social and economic sciences with engineering education is essential to achieving these goals.

The community at large demands a transition to sustainable solutions. As the energy and materials sectors enter a transformative stage, trained professionals proficient in STEM (science, technology, engineering, and mathematics) disciplines, but also able to consider societal needs, will be highly sought after to make informed decisions in concert with the wide community. Stanford University has announced the launch of the School of Sustainability,^178^ and several institutions are developing interdisciplinary master’s-level programs, for example, the M.Sc. in Global Management of Natural Resources, offered by University College London,^179^ which developed from the European research consortium ShaleXenvironmenT,^180^ and the M.S. in Sustainability: Energy and Materials Management, soon to be offered by the University of Oklahoma.

## Concluding Remarks

Much research and innovation has been embedded in the energy and materials industries since their inception, enabling their lasting success. What will then be the different impetus that will enable these sectors to embrace the transformation imposed by the current, fast-transforming socioeconomic landscape? In the authors’ opinion, three aspects are critical:

1. Within each stage of Figure 5, research will enable innovative sustainable and renewable solutions. This ranges from more renewable energy in the “production” stage, to the manufacture of new chemicals and materials that will enable fast cooling of electrochemical devices (e.g., batteries) to efficiently store renewable solar and wind energy, notoriously intermittent, in the “derivatives” stage, etc.

2. A few, selected, research needs are listed in Table 1. The success of these research propositions depends on the integration of experimental and computational techniques. Machine learning is becoming attractive and effective; once provided with sufficiently large and reliable data sets, ML will enable fast progress. Nevertheless, in our opinion, a fundamental understanding of the fundamental mechanisms responsible for macroscopic observations remains essential for achieving transformative solutions.

3. As both the energy and the chemicals sectors seek to become more sustainable, opportunities will open up for embedding the cradle-to-cradle approach in the design of new products, materials, and processes. A few examples are provided in Table 2. The identification of sustainable solutions is only possible when an analysis is conducted within the life cycle of a technology, a product, or a service, which encompasses economic, environmental, and societal footprints. Such comprehensive analysis is becoming essential for achieving and maintaining the social license to operate.

Although the challenges ahead might seem formidable, chemical engineers are able to identify opportunities in challenging times. Any successful effort toward achieving a sustainable future will lead to spectacular benefits for the whole society. Different solutions might be optimal in different geopolitical and social environments. This could lead to bespoke, localized solutions, which could pose an additional challenge to integrated industries as we know them today. To facilitate the transition, the environmental, social, and economic impacts of these local solutions should be carefully assessed. Certainly, education will play an enormous role. Because future challenges encompass technical, social, economic, and environmental aspects, training should provide seamless integration of these disciplines, perhaps via interdisciplinary undergraduate, master’s, and Ph.D. level programs, which will bring to fruition the vision Prausnitz shared at the beginning of the 21st century.
